# The role of lung ultrasonography in the assessment of overhydration in maintenance hemodialysis patients

**DOI:** 10.1080/0886022X.2022.2132169

**Published:** 2023-01-17

**Authors:** Linlin Cui, Jiejian Chen, Chaoyang Ye

**Affiliations:** a Kidney Institute of CPLA and Division of Nephrology, Changzheng Hospital, Naval Military Medical University, Shanghai, China; b Department of Nephrology, The 900th Hospital of Joint Logistic Support Force, PLA, Fuzhou, China; c Department of Nephrology, Shuguang Hospital Shanghai University of Traditional Chinese Medicine, Shanghai, China

**Keywords:** Lung ultrasonography, hemodialysis, excessive fluid volume, mortality

## Abstract

**Purpose:**

Existed methods like biochemical markers improve the accuracy of fluid evaluation for the maintenance hemodialysis patients, but none of them has become the gold standard. This study aimed to evaluate the potential of lung ultrasonography as a useful tool for monitoring the volume status of the patients.

**Methods:**

A total of 88 patients undergoing maintenance hemodialytic were enrolled in this prospective observational study. Patients were divided into three groups: overhydration (OH), normohydration, and hypohydration according to bioimpedance spectroscopy. Lung ultrasonography parameters, echocardiography parameters, and clinical characteristics of three groups were analyzed. After an average follow-up of 433 days, all-cause mortality among groups was compared.

**Results:**

The total number of lung comets was statistically reduced in patients after dialysis (*Z*= −6.891, *p* < 0.001). This reduction was related to ΔOH (OH – Δ*W* (the weight gain from dry weight)) and echocardiographic parameters, which proved the relationship among the comet-tail, hydration status of body and cardiac performance. The Kappa consistency test showed that lung ultrasonography and bioelectrical spectroscopy had moderate consistency. ROC analysis showed that the best cut-point of lung comet is 13. The pre-/post-dialysis lung comet-tail, cardiac function and total body impedance with all-cause mortality was investigated. Kaplan–Meier’s analysis revealed that the all-cause mortality was higher in lung congestion patients.

**Conclusions:**

This study proposes a potentially reliable lung ultrasonography method for estimating fluids overload, which also has implication value of all-cause mortality.

## Introduction

Dialysis adequacy contains the elimination of toxins and fluid, is one of the most important objectives in end-stage kidney disease (ESKD) patients. Although much progress has been made in toxin assessment, there are still significant limitations in liquid assessment. The hypohydration causes hypotension and the overhydration (OH) is directly associated with left ventricular hypertrophy, both of which can result in high mortality and morbidity of cardiovascular disease in ESKD patients [1–3]. Currently, the ‘dry weight’ is used to name the standard weight in patients with maintenance hemodialysis, which means the lowest weight they can tolerate without symptoms of OH and hypohydration. However, many nephrologists in most dialysis centers assess the ‘dry weight’ based on clinical symptoms and signs. This subjective clinical method is imprecise and does not consider nutritional status. When the setting is higher than the actual ‘dry weight’, maintenance hemodialysis patients are characterized by a condition of volume overload, thereby leading to refractory hypertension, left ventricular hypertrophy and congestion heart failure [4,5]. On the contrary, they may be characterized by a state of hypohydration, such as hypotension, muscle twitches, vomit, and so on [2].

Several techniques have been used in clinic and have shown great impact on fluid evaluation, including biochemical markers, blood volume monitoring, vena cava diameter, bioimpedance spectroscopy, etc. [1,6,7]. All these methods may improve the accuracy of fluid evaluation, but none of them has become the gold standard. Lung ultrasonography has recently been proved to be useful for defining extravascular lung water (ELW), which is a relatively small but basic component of total body fluid. ELW represents the fluid content of the lung interstitium and it is closely associated with the filling pressure of the left ventricle. Therefore, lung ultrasonography examination of interstitial imbibitions or pleural effusion, decompensated heart failure, and rapid fluid clearance in maintenance hemodialysis patients has received more and more attention in clinical research [8–10]. The aim of this study was to evaluate the functional role of lung ultrasonography in detecting total body fluid and predicting the all-cause mortality in ESKD patients. Thus, we performed the lung ultrasonography combined with bioimpedance spectroscopy and echocardiography in a group of maintenance hemodialysis patients of Shanghai, China, in a cohort from a single hemodialysis unit.

## Participants and methods

### Participants

Eighty-eight patients who received maintenance hemodialytic treatment were recruited at the Dialysis Unit of Shanghai Changzheng Hospital from 1 July 2014 to 30 June 2015. Hemodialysis therapy was performed using Fresenius FX 80 dialyzer (1.5 m^2^; Fresenius Medical Care, Bad Homburg, Germany) for 4 h, three times a week. Biochemical parameters were determined on the first Monday or Tuesday of each month, per-dialysis, after the long interval of dialysis. All participants were informed and signed the consent. All protocols were carried out following the principles of the Declaration of Helsinki, and authorized by the Ethics Committee of Shanghai Changzheng Hospital (20211226).

### Inclusion and exclusion criteria

The inclusion criteria: (1) the patients who had received long-term hemodialysis treatment for at least 3 months; (2) over 18 years old; (3) the patients who had received standard bicarbonate dialysis three times a week. The exclusion criteria: (1) pulmonary disease: such as interstitial lung disease, severe pneumonia, pulmonary tumor, and acute severe pulmonary edema; (2) cardiac disease: organic cardiopathy/severe congestive heart failure; (3) metallic joint prostheses, cardiac pacemakers or stents; (4) limb amputations or limb skin defect; (5) other electrical examinations were in progress, such as ambulatory blood pressure monitoring.

### Procedures and evaluations

**Figure 1.**
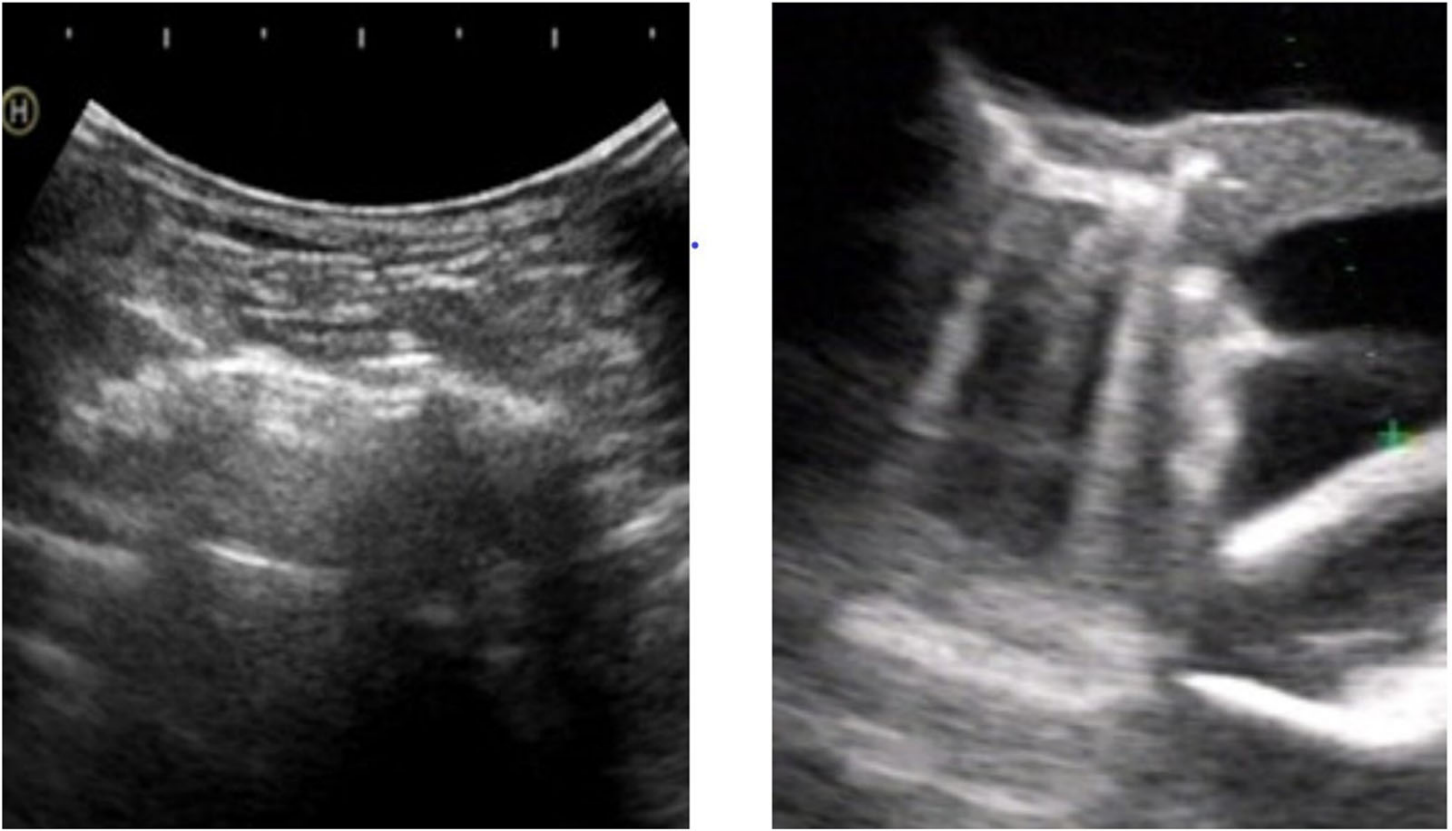
The ultrasonography of normal lung and pulmonary congestion. (Left) Normal lung ultrasonography: the regular, parallel, horizontal hyperechogenic lines are due to the lung-wall interface. (Right) Pulmonary congestion: lung comets are hyperechogenic, coherent vertical bundles with narrow basis that extends from the transducer to the further border of the screen.

The lung ultrasonography examination was started 30 min before and after dialysis. The examination was performed utilizing the echographic equipment with 3.0 MHz convex probe, and the patients were in the supine or semisupine positions. The main scanning area is the second to fifth intercostal space on the right side, the side of the breastbone to the mid-axillary line, and the second to fourth intercostal space on the left side. The comet-tail sign was defined as the wedge-shaped echogenic artifact, and from the transducer to the limit of the screen (Figure 1). At each scan site, the signs of comet-tail were calculated, and the sum of these signs was a score, which represents the degree of extravascular fluid in the lung.

In the above 28 regions, the comet-tail signs were counted and the sum of the signs was a score, which denoting the degree of extravascular fluid in the lung.

The total body water (TBW) volume was estimated by bioimpedance spectroscopy apparatus (body composition monitor; Fresenius Medical Care, Bad Homburg, Germany) 30 min before dialysis and 30 min after dialysis. The electrodes were connected to the non-fistula forearm and ipsilateral ankle of patients, and the patients were in supine or semi-supine positions. The multi-frequency bioimpedance spectroscopy can distinguish the resistance among the TBW, the extracellular water (ECW), and the intracellular water (ICW). Then, TBW, ECW, ICW, and OH were determined through mathematical modeling. To facilitate the comparison among the patients, the OH that normalized to interdialytic weight gain (ΔOH = OH – Δ*W*) was determined. Then patients were divided into three groups: OH (ΔOH > 1), normohydration (–1 ≤ ΔOH ≤ 1) and hypohydration (ΔOH< −1).

Echocardiographic measurement was performed 30 min before dialysis treatment, the following indicators were measured: ejection fractions, fractional shortening, fractional shortening, left atrial volume, and left ventricular end-diastolic volume.

### Statistical analysis

Data were presented as mean ± standard deviation (SD), or median and interquartile range. Normally distributed data were analyzed by *t*-test, paired *t*-test or ANOVA test, and non-normally distributed data were analyzed by Wilcoxon’s signed rank test. Significance for associations was analyzed by Pearson’s correlation or Spearman’s correlation. The agreement between categorical variables was measured by kappa statistics. The receiver operating characteristic curve (ROC) analysis was performed to evaluate the feasibility of the lung ultrasonography in assessing body water volume. The Cox regression analysis and Kaplan–Meier’s analysis were used to investigate the prognostic value of the lung ultrasound in predicting mortality. *p* < 0.05 was considered as statistically significant.

## Results

This study enrolled a total of 88 patients (52.27% males) who underwent maintenance hemodialysis treatment from 1 July 2014 to 30 June 2015. The average blood pressure of all patients before dialysis was 155.94 ± 27.57/84.47 ± 16.46 mmHg. Among the participants, there were 42 diabetic patients, 24 smokers and 21 anuria patients (24-h urine volume, 0–100 mL). Eighteen patients presented pedal edema before dialysis, and 21 patients were treated with no less than three antihypertensive drugs (angiotensin-converting enzyme inhibitors (ACEIs)/angiotensin receptor blockers (ARBs), calcium-channel blockers (CCBs), b-blocker or a-blocker) at the same time. In addition, according to the New York Heart Association (NYHA) scale, the cardiac function of patients was analyzed, and the patients were classified as symptomatic or asymptomatic. Among them, 23 patients had a NYHA score ≥3. The mean weight gain from dry weight was 1.71 ± 1.06 kg. The causes of ESKD included glomerulopathies (27.3%), diabetes (27.3%), hypertension (15.9%), polycystic kidney disease (6.8%), nephrolithiasis (4.5%), multiple myeloma (3.4%), pyelonephritis (2.3%), ischemic nephropathy (1.1%), neoplasms (1.1%), nephrotuberculosis (1.1%), and unknown (9.1%).

### Pre-dialysis

The patients were divided into three groups according to ΔOH: OH (ΔOH > 1), normohydration (–1 ≤ ΔOH ≤ 1) and hypohydration (ΔOH< −1). The demographic and clinical characteristics of the study population are summarized in Table 1. Compared with normohydration and hypohydration patients, there were more OH patients with 24-h urine volume (625 mL; *p* < 0.001) and pedal edema (*p* = 0.004). The mean weight gain from dry weight of OH patients was less (*p* = 0.004) and dialysis vintage was shorter (*p* = 0.011). The albumin and hemoglobin content of OH patients was significantly lower than that of normohydration and hypohydration patients (*p* < 0.001, *p* = 0.003). There were significantly more lung comets in OH patients than that in normohydration and hypohydration patients (*p* < 0.001). However, there was no significant difference in lung comets between normohydration patients and hypohydration patients.

**Table 1.** Main demographic and clinical characteristics of the study population.

	ΔOH<–1 (*n* = 16)	–1 ≤ ΔOH ≤ 1 (*n* = 24)	ΔOH > 1 (*n* = 48)	*p* Value
Age, years	57.9 ± 16.5	65.5 ± 13.8	58.4 ± 15.5	0.148
Male, %	50.0	33.3	62.5	0.064
BMI, kg/m^2^	23.7 ± 3.5	24.4 ± 3.8	23.1 ± 3.0	0.349
Mean weight gain (Δ*W*), kg	2.2 ± 0.9	2.0 ± 1.3	1.4 ± 0.9	0.004
Diabetes, %	31.3	41.7	56.3	0.174
Smokers, %	25.0	12.5	35.4	0.117
Arteriovenous fistula, %	81.3	66.7	77.1	0.513
Pedal edema, %	0.0	8.3	33.3	0.004
Systolic pressure, mmHg	149.7 ± 35.0	152.9 ± 28.8	159.5 ± 24.1	0.385
Diastolic pressure, mmHg	84.4 ± 19.7	81.3 ± 15.7	86.1 ± 15.8	0.504
Albumin, g/L	41.4 ± 3.6	39.1 ± 5.0	35.5 ± 5.8	<0.001
Hemoglobin, g/L	116.1 ± 24.7	107.7 ± 21.1	94.7 ± 23.1	0.003
Calcium, mmol/L	2.4 ± 0.3	2.3 ± 0.3	2.3 ± 0.3	0.297
Phosphate, mmol/L	1.8 ± 0.5	1.6 ± 0.4	1.5 ± 0.5	0.089
Dialysis vintage, months	27 (12, 84)	24 (6, 36)	11 (3, 24)	0.011
24-hour urine volume, mL	100 (100, 425)	250 (100, 625)	625 (300, 1000)	<0.001
Lung comet	1 (0, 6.25)	1.5 (0, 5.75)	24.76 (5.25, 37.58)	<0.001

A total of 74 patients successfully completed the echocardiographic measurements. We analyzed the correlation between lung comet and echocardiographic parameters. Lung comet was negatively correlated with ejection fractions (*r*= −0.509, *p* < 0.001), fractional shortening (*r*= −0.303, *p* = 0.009), and positively correlated with left ventricular end-diastolic volume (*r* = 0.345, *p* = 0.003) and left atrial volume (*r* = 0.419, *p* < 0.001) (Table 2).

**Table 2.** The relationship between the lung comet and echocardiographic parameters.

	Mean ± SD	Pre-dialysis		Pre-/post-dialysis	
		*r*	*p* Value	*r*	*p* Value
Ejection fractions	0.58 ± 0.09	–0.509	<0.001	–0.427	<0.001
Fractional shortening	0.30 ± 0.09	–0.303	0.009	–0.344	0.003
Left atrial volume	38.57 ± 6.14	0.419	<0.001	0.355	0.002
Left ventricular end-diastolic volume	51.89 ± 5.61	0.345	0.003	0.324	0.005

### Post-dialysis

We adjusted the hemodialysis ultrafiltration of all patients according to the ΔOH. The mean ultrafiltration volume during dialysis was 2.03 ± 0.96 kg. After dialysis, the proportion of normohydration patients increased from 24 (26.14%) to 30 (34.09%). The arterial pressure of the patients after dialysis was 141.19 ± 27.63/79.96 ± 13.08 mmHg, which was significantly different from that pre-dialysis (*p* < 0.001, *p* = 0.039). TBW and ECW after dialysis were significantly reduced from pre-dialysis (*p* < 0.001, *p* < 0.001), while there was no significant difference about ICW (*p* = 0.551). We observed a statistically significant reduction in the total number of lung comets in patients after dialysis (*Z*= −6.891, *p* < 0.001). The lung comet in OH patients was 7.5 (1.25, 13), which was significantly higher than that in normohydration [5 (0, 10)] and hypohydration [0 (0, 2)] patients (*p* < 0.001).

### Pre- and post-dialysis

Next, we found that the reduction in the lung comet score was not correlated with the ultrafiltration volume, but was significantly correlated with the pre-dialysis ultrasound lung comets (ULC) score (*r* = 0.955, *p* < 0.001) and ΔOH (*r* = 0.527, *p* < 0.001). The decreased lung comet score was negatively correlated with pre-dialysis ejection fractions (*r*= −0.427, *p* < 0.001), fractional shortening (*r*= −0.344, *p* = 0.003), and positively correlated with left ventricular end-diastolic volume (*r* = 0.324, *p* = 0.005) and left atrial volume (*r* = 0.355, *p* = 0.002) (Table 2).

### The consistency between lung ultrasonography and bioimpedance spectroscopy

The Kappa consistency test was utilized to evaluate the consistency between lung ultrasonography and bioimpedance spectroscopy in all patients and non-peripheral edema patients. These patients were classified into two parts according to the comet tail sign: absent or mild pulmonary congestion (comet tail sign <14) and severe pulmonary congestion (comet tail sign ≥14). Furthermore, these patients were also classified into two parts according to bioimpedance spectroscopy apparatus: normohydration (|ΔOH|≤1) and non-normohydration (|ΔOH|>1). In all patients group, Kappa consistency test showed that lung ultrasonography and bioelectrical spectroscopy had moderate consistency (Kappa = 0.621, *p* < 0.001), while the patients without obvious peripheral edema showed better consistency (Kappa = 0.706, *p* < 0.001).

**Figure 2.**
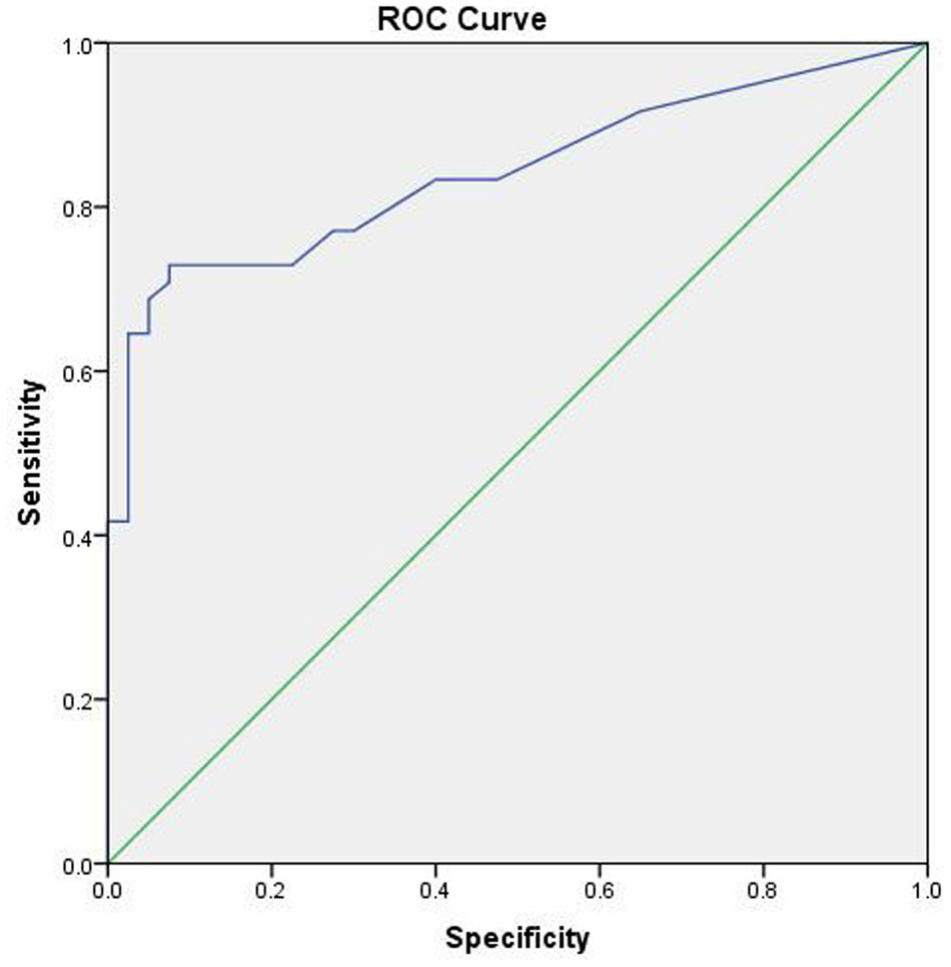
The ROC curve of lung ultrasonography. The ROC analysis shows high accuracy (AUC: 0.841 (0.758–0.925)) in detecting fluids overload. When the cut-point of lung comet is 12.5, the sensitivity (0.688) and specificity (0.950) of lung ultrasonography for detecting the fluid overload are the best.

According to the ROC analysis, the comet tail sign may be an indicator for distinguishing clinically significant OH patients from non-hyperhydration patients, and identifying the degree of mild and severe pulmonary congestion. The ROC analysis revealed high accuracy (area under roc curve (AUC): 0.841 (0.758–0.925)) in detecting fluids overload (Figure 2). When the cut-point of lung comet was 13, the sensitivity (0.660) and specificity (0.950) of lung ultrasonography for detecting the fluid overload were the best.

### Survival

**Figure 3.**
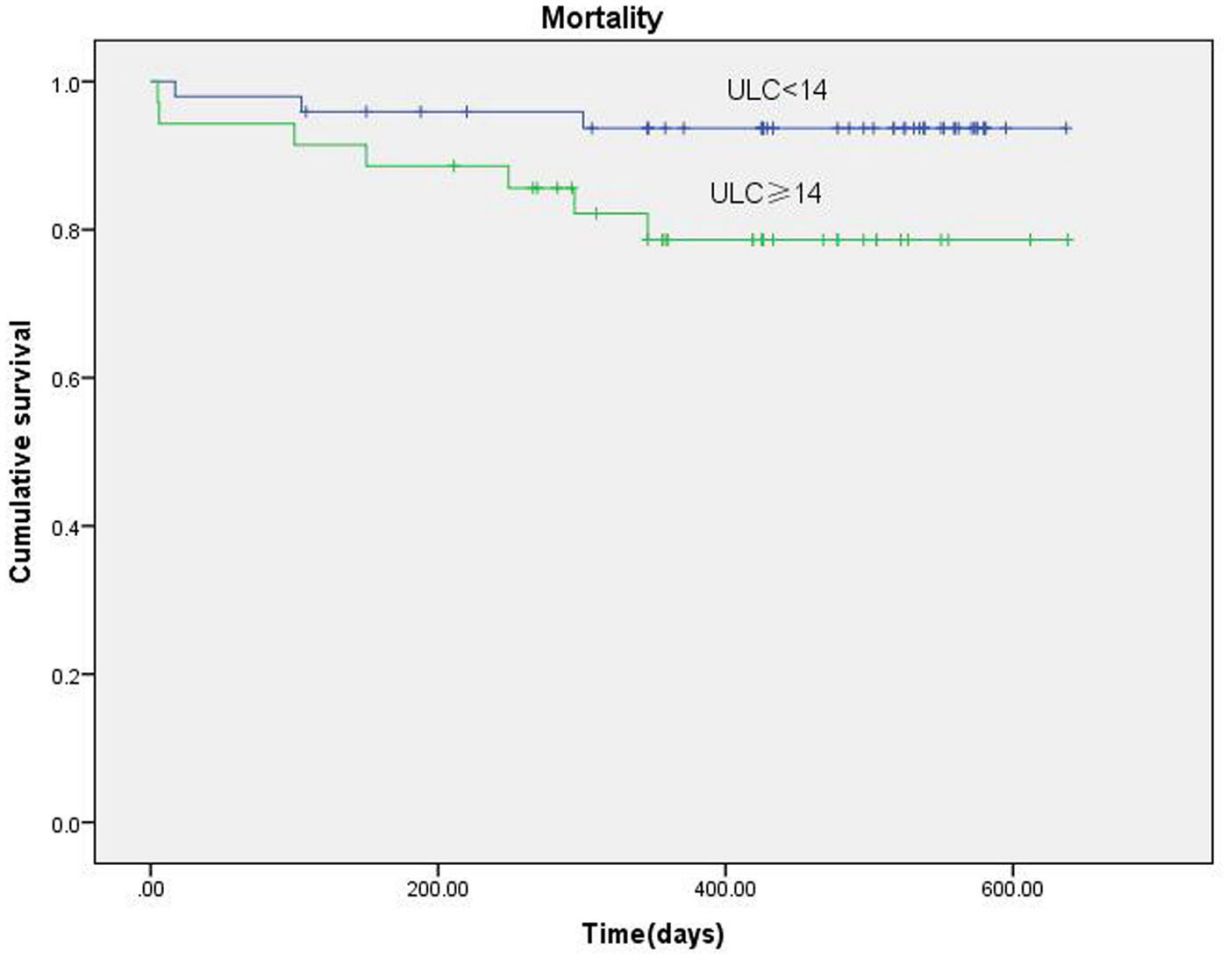
Kaplan–Meier’s survival analysis according to the lung comet-tail. Kaplan–Meier’s analysis shows that patients with severe lung congestion have a higher mortality than patients with absent or mild lung congestion (*p*= 0.048).

During the follow-up period, 10 patients died and four patients were lost to follow-up. The median of observation time was 433 (346, 538) days. Kaplan–Meier’s analysis showed that patients with severe lung congestion had a higher mortality than patients with absent or mild lung congestion (*p* = 0.048) (Figure 3). Furthermore, the Cox regression analysis was used to investigate the risk factors of death. The Cox regression showed that the comet-tail score was a risk factor for death in dialysis patients (OR = 3.909, *p* = 0.048). Then, we used a multivariate Cox model, which included the comet-tail score, NYHA class, hydration status, basal epidemiological (age, sex, BMI, edema, diabetes, vascular access, and so on) and some lab data (hemoglobin, albumin, and phosphorus). The results showed that age (OR = 1.157, *p* = 0.010) maintained a significant correlation with survival time (Table 3). While comet-tail score (OR = 7.704, *p* = 0.076) and hydration status (OR = 5.339, *p* = 0.390) had no statistical significance.

**Table 3.** Survival of the study population.

	OR (95%CI)	*p* Value
Age, years	1.157 (1.036–1.292)	0.010
ULC score (0= <14, 1= ≥14)	7.704 (0.810–73.284)	0.076
Hydration status (1 = over, 2 = norm and 3 = hypo)	5.399 (0.116–251.792)	0.390
NYHA class (0 = I–II, 1 = III–IV)	0.567 (0.061–5.225)	0.616
Male	1.012 (0.096–10.675)	0.992
BMI, kg/m^2^	0.839 (0.640–1.099)	0.202
Diabetes	0.245 (0.025–2.423)	0.229
Smokers	0.622 (0.057–6.791)	0.697
Arteriovenous fistula	2.042 (0.248–16.831)	0.507
Pedal edema	0.751 (0.027–10.896)	0.866
Systolic pressure, mmHg	1.018 (0.970–1.068)	0.472
Diastolic pressure, mmHg	1.009 (0.952–1.070)	0.760
Albumin, g/L	1.174 (0.982–1.405)	0.079
Hemoglobin, g/L	1.007 (0.962–1.055)	0.755
Phosphate, mmol/L	0.279 (0.032–2.433)	0.248

CI: confidence interval; NYHA: New York Heart Association; OR: odd ratio; ULC: ultrasound lung comets.

## Discussion

The lung ultrasonography has been considered as a highly sensitive method for detecting pulmonary edema in dyspnea patients [11,12]. The interlobular septum thickens under lung congestion, and then the ultrasound beam is reflected at the septum. The reflection of the beam creates the phenomenon of lung comets [13]. Due to volume overload, dialysis patients often experience lung imbibitions similar to pulmonary edema. Therefore, we believe that this principle can also apply to maintenance hemodialysis patients. The lung ultrasonography may be a potentially useful tool for monitoring the volume status of maintenance hemodialysis patients, which have been roughly assessed by traditional methods in the past.

The main findings of this study were: (1) comet tails was significantly reduced after hemodialysis. This reduction was significantly related to ΔOH and echocardiographic parameters, demonstrating the direct relationship among the comet-tail, hydration status of body and cardiac performance. Mallamaci et al. have also reported that the rapid reduction of the lung comet is only closely related to the altered left ventricle performance, but scarcely associated with hydration status [14]. Donadio et al. have reported the opposite result, that is, the dynamic change of B-lines is correlated with extracellular, but has nothing to do with intra-cellular water index [15]. However, Siriopol et al. have demonstrated that the reduction is not correlated with any echocardiographic or bioimpedance parameters [10]. ELW is known as a relatively small but fundamental component of total body fluid, and is associated with the filling pressure of the left ventricle. Therefore, the lung comet depends on cardiac function and circulating volume. When the heart function is normal, lung comet is the best hemodynamic parameter reflecting the volume of circulation. (2) We compared the performance of lung ultrasound and bioimpedance to assess volume status to better clarify the relationship between the different methods. The Kappa consistency test showed that the lung ultrasonography and bioelectrical spectroscopy had moderate consistency. Considering that patients with peripheral edema were able to distinguish volume overload or not, we also performed a subgroup Kappa consistency test in patients without peripheral edema. Surprisingly, the subgroup showed better consistency. Furthermore, bioelectrical spectroscopy showed that the best cut-point for lung comets was 13. So far, a gold standard for dry weight assessment has not yet been established. Therefore, we have to choose the bioimpedance as a comparison method in this study, which has been used clinically for many years [1,16–18]. It distinguishes TBW, ECW, and ICW by injecting electric current into the body, and finally defines the individual hydration status through certain mathematical modeling. As a consequence, it does not take heart function into consideration. That may be the main reason for moderate consistency between lung ultrasonography and bioelectrical spectroscopy. (3) A implication value of all-cause mortality. This work holistically investigated the pre-/post-dialysis lung comet-tail, cardiac function and total body impedance with all-cause mortality. Despite the negative reactions in the multivariate Cox model, lung congestion patients had a higher all-cause mortality, which opened up promising avenues for future trials. Dialysis patients had a greater risk of all-cause mortality as compared with patients without kidney disease. Siriopol et al. have enrolled 96 patients from a single hemodialysis unit and followed up for a median of 405.5 days [10]. The mortality of severe lung congestion (>30) is higher than that of the other groups, and the lung comet score before hemodialysis has a significant discriminating power for survival. Zoccali et al. also have tested the prognostic value of the lung comet for hemodialysis patients, showing that patients with very severe lung congestion (>60) have a higher risk of cardiac events and all-cause death [19]. In an international, multi-center randomized controlled trial, Zoccali et al. investigated a lung ultrasound-guided treatment could improve recurrent episodes of decompensated heart failure and cardiovascular events [20]. In our research, the Kaplan–Meier analysis showed that patients with severe lung congestion had a higher mortality than patients with absent or mild lung congestion. The Cox regression showed that the comet-tail score was a risk factor for death in dialysis patients (OR = 3.909, *p* = 0.048). However, the multivariate Cox model which included the comet-tail score, NYHA class, hydration status, basal epidemiological (age, sex, BMI, edema, diabetes, vascular access, and so on) and some lab data (hemoglobin, albumin, and phosphorus), showed that only age (OR = 1.157, *p* = 0.010) maintained a significant correlation with survival time. The main reasons of negative respond in the multivariate Cox model might be the most enrolled patients were absent or mild lung congestion, and the follow-up period was shorter.

## Limitation

The limitations of our study were the small number of enrolled patients from a single dialysis center and the short follow-up period. Moreover, the lung ultrasonography only provided information about OH, not information about hypohydration. The lung comet in MHD patients was not fixed and it could be differed through enhancing ultrafiltration. It would be more reasonable to monitoring with serial measurements. Finally, we did not collect the non-fatal events and specify the cause of death. Therefore, more large-scale studies are needed to evaluate the value of lung ultrasonography. The cutoff for diagnosis of volume overload also needs to be ensured in following studies.

## Supplementary Material

Supplemental Material

## Data Availability

All data generated or analyzed during this study are included in this article or its supplementary material files. Further enquiries can be directed to the corresponding author.
