# Supplementary material for: The role of lung ultrasonography in the assessment of overhydration in maintenance hemodialysis patients
Source: Ren Fail. 2023 Jan 17;44(1):1985–92. doi: 10.1080/0886022X.2022.2132169 (PMC9848227; doi:10.1080/0886022X.2022.2132169)
Supplement: Supplemental Material [file IRNF_A_2132169_SM3135.zip › IRNF 2132169/Additional_file_1.pdf]

Table S1. Baseline characteristics and outcomes in AKI and non-AKI patients with sepsis

Characteristics	Non-AKI (n=864)	AKI (n=1211)	P value
Age, Mean (SD)	61.4 (17.9)	63.4(17.8)	<0.001
Sex, male (%)	560 (64.8)	796 (65.7)	0.666
Body mass index, n (%)			<0.001
<18.5	62 (7.2)	96 (7.9)	
18.5-24.9	609 (70.5)	774 (63.9)	
25.0-27.9	154(17.8)	219 (18.8)	
≥28.0	39 (4.5)	122 (10.1)	
Baseline SCr, µmmol/L	64.7 (50.3-82.9)	67.2 (56.0-99.0)	0.045
Severity on admission			
APACHEII, median (IQR)	18.0 (13.0-23.0)	21.0 (15.0-27.0)	<0.001
SOFA, median (IQR)	6.0 (3.0-8.0)	8.0 (5.0-11.0)	<0.001
Source of admission, n (%)			0.853
Emergency Department	204 (23.6)	286 (23.3)	
General Wards	310 (35.9)	450 (37.2)	
Emergency Surgery	118 (13.7)	157 (13.0)	
Selective Surgery	70 (8.1)	81 (6.7)	
Others	161 (18.8)	237 (19.8)	
Comorbidities, n (%)			
Respiratory disease	76 (8.8)	87 (7.2)	0.178
Cardiovascular disease	101 (11.7)	216 (17.8)	<0.001
Hypertension	251 (29.1)	443 (36.3)	<0.001
Diabetes mellitus	154 (17.8)	250 (20.6)	0.110
Chronic kidney disease	25 (2.9)	111 (9.2)	<0.001
Cancer	91 (10.5)	116 (9.6)	0.475
None	257 (29.7)	319 (26.3)	0.088
Treatment during ICU, n (%)			
Mechanical ventilation	647 (74.9)	967 (79.7)	0.007
Renal replacement therapy	48 (5.6)	389 (32.1)	<0.001
Vasopressor	139 (16.1)	739 (61.0)	<0.001
FB first 24 h, median (IQR), L	1.4 (-0.1-2.9)	3.1 (1.1-4.6)	<0.001
Outcomes			
In-hospital mortality	224 (25.9)	463 (38.2)	<0.001
In-ICU mortality	180 (20.8)	387 (32.0)	<0.001
ICU length of stay,	6.0 (4.0-15.0)	8.0 (5.0-17.0)	0.001
Hospital length of stay	18.0 (10.0-28.0)	18.0 (10.0-28.0)	0.837

Table S2. Baseline characteristics and outcomes for subgroup patients in BAKIT according to the timing onset and evolution of AKI

Characteristics	Early transient AKI	Early persistent AKI	Late transient AKI	Late persistent AKI	P value
Number	118	305	90	140	
Age, Mean (SD)	68.4 (17.5)	70.0 (16.5)	66.6 (17.6)	65.9 (18.4)	0.032
Sex, male (%)	79 (66.9)	195 (63.9)	54 (60.0)	86 (61.4)	0.715
Baseline SCr, mean (SD),					
Body mass index, n (%)					
< 18.5	19 (16.1)	64 (21.0)	5 (5.6)	14 (10.0)	<0.001
18.5-24.9	67 (56.8)	153 (50.2)	61(67.8)	88 (62.9)	
25.0-27.9	13 (11.0)	14 (4.6)	15 (16.6)	25 (17.9)	
≥ 28.0	19 (16.1)	74 (24.3)	9 (10.0)	13 (9.3)	
Source of admission, n (%)					
Emergency Department	40 (33.9)	109 (35.7)	44 (48.9)	46 (32.9)	0.001
Wards	38 (32.2)	130 (42.6)	26 (28.9)	44 (31.4)	
Postoperative care	37 (31.4)	48 (15.7)	16 (17.8)	40 (28.6)	
Others	3 (2.5)	18 (5.9)	4 (4.4)	10 (7.1)	
Comorbidities, n (%)					
COPD	13 (11.0)	21 (6.9)	12 (13.3)	8 (5.7)	0.103
Cardiovascular disease	38 (32.2)	115 (37.7)	31 (34.4)	43 (30.7)	0.472
Hypertension	48 (40.7)	151 (49.5)	40 (44.4)	62 (44.3)	0.375
Diabetes mellitus	22 (18.6)	80 (26.2)	14 (15.6)	41 (29.3)	0.040
Chronic kidney disease	5 (4.2)	53 (17.4)	5 (5.6)	32 (22.9)	<0.001
Cancer	31 (26.3)	54 (17.7)	13 (14.4)	22 (15.7)	0.086
None	26 (22.0)	57 (18.7)	32 (35.6)	37 (26.4)	0.015
Source of infection, n (%)					
Hospital-acquired	55 (46.6)	141 (46.2)	39 (43.3)	59 (42.1)	0.832
Community-acquired	63 (53.4)	161 (53.8)	59 (56.7)	81 (57.9)	
Site of infection[#], n (%)					

Lung	62 (52.5)	165 (54.1)	51 (56.7)	95 (67.9)	0.033
Abdominal	27 (22.9)	114 (37.4)	21 (23.3)	31 (22.1)	0.001
Urinary	6 (5.1)	37 (12.1)	4 (4.4)	8 (5.7)	<0.001
Bloodstream	8 (6.8)	29 (9.5)	5 (5.6)	11 (7.9)	0.598
Others	7 (5.9)	11 (3.6)	5 (5.6)	9 (6.4)	0.542
Unclear	13 (11.0)	57 (18.7)	8 (8.9)	15 (10.7)	0.024
AKI stage, n (%)					
1	60 (50.8)	46 (15.1)	49 (54.4)	30 (21.4)	
2	29 (24.6)	75 (24.6)	26 (28.9)	40 (28.6)	<0.001
3	29 (24.6)	184 (60.3)	15 (16.7)	70 (50.0)	
Outcomes					
MAKE, n(%)	61 (51.7)	242 (79.3)	21 (23.3)	81 (57.9)	<0.001
Hospital mortality, n(%)	46 (39.0)	183 (60.0)	17 (18.9)	55 (39.3)	<0.001
ICU LOS, days, median (IQR)	10.0 (6.0 - 17.0)	10.0 (5.0 - 19.0)	8.0 (4.0- 17.0)	9.0 (4.0- 16.0)	0.005
Hospital LOS, days, median (IQR)	22.0 (14.0- 34.0)	24.0 (9.0 - 36.0)	20.0 (13.0- 29.0)	22.0 (16.0- 37.0)	<0.001

Table S3. Symptoms, clinical characteristics for subgroup patients in BAKIT according to the timing onset and evolution of AKI

Values	Early transient AKI	Early persistent AKI	Late transient AKI	Late persistent AKI	P value
Temperature ≥ 38℃	40 (34.8)	121 (40.5)	40 (46.0)	56 (40.3)	0.454
Heart rate /min, mean (SD)	108.8 (22.9)	117.1 (25.4)	101.6 (20.4)	117.7 (25.2)	<0.001
>100	61 (52.6)	203 (68.1)	21 (23.3)	97 (70.3)	<0.001
Respiratory rate/min, mean (SD)	25.3 (4.1)	26.1 (4.7)	24.2 (3.9)	25.1 (4.3)	0.020
>20	63 (53.4)	198 (64.9)	49 (54.4)	81 (57.9)	0.084
MAP < 65 mmHg, n (%)	47 (42.0)	168 (56.0)	18 (20.2)	71 (51.4)	<0.001
Glasgow Coma Scale < 10, n (%)	40 (33.9)	177 (58.0)	21 (23.3)	40 (28.6)	<0.001
Laboratory findings, n (%)					
WBC ($\times 10^9$/L), median (IQR)	13.0 (9.0 - 17.7)	13.3 (9.3 - 18.3)	12.4 (8.3 - 18.2)	13.2 (7.7 - 17.6)	0.026
HCT < 30%	37 (31.4)	184 (60.3)	22 (24.4)	78 (57.7)	<0.001
Platelet < 100×10^9/L, n (%)	36 (30.8)	140 (40.6)	15 (16.7)	44 (31.7)	< 0.001
BIL > 23.3 mmol/L, n (%)	17 (15.2)	83 (28.7)	20 (23.0)	43 (33.9)	0.007
pH, mean (SD)	7.34 (0.11)	7.31 (0.12)	7.37 (0.11)	7.33 (0.11)	< 0.001
<7.32	42 (35.6)	161 (53.0)	23 (25.6)	62 (44.6)	<0.001
PO_2/FIO_2, median (IQR)	225.0 (115.9 - 298.0)	197.0 (140.0 - 283.0)	225.5 (175.0 - 320.0)	224.5 (147.8 - 302.0)	0.024
< 200 mmHg, n (%)	37 (34.3)	147 (51.0)	28 (32.6)	54 (40.9)	0.002
PCO_2, mean (SD)	40.0 (33.0 - 47.9)	36.0 (30.0 - 44.0)	37.0 (32.8 - 43.3)	35.0 (29.6 - 44.0)	0.010
Fluid balance, day1, L	2.0 (1.5 - 3.6)	3.1 (1.9 - 4.6)	1.7 (0.5 - 2.6)	2.9 (1.6 - 4.6)	<0.001

Table S4. Multivariate logistic regression analysis evaluating the risk factors on early persistent AKI in BAKIT

Characteristics	OR(95%CI)	p
Age, per year older	1.02 (1.00 - 1.03)	<0.001
Body mass index, kg/m^2		
< 18.5	2.56 (1.17 - 4.33)	0.015
18.5-24.9	Ref	
25.0-27.9	1.27 (0.77 - 2.08)	0.276
≥ 28.0	2.65 (1.48 - 3.26)	<0.001
Heart rate, per 5/min	1.02 (1.01 - 1.06)	0.036
GCS < 10	2.14 (1.34 - 3.41)	0.002
Mean arterial pressure < 65 mmHg	1.61 (1.16 - 2.45)	0.007
Platelet< $100000/cm^3$	1.47 (1.09 - 2.25)	0.001
Hematocrit < 30%	1.53 (1.26 - 2.04)	0.005
pH <7.32	1.07 (1.04 - 1.69)	0.032
PO_2/FIO_2 < 200 mmHg	1.89 (1.20 - 3.00)	0.006
Fluid balance day 1, per L/24 h	1.78 (1.26 - 2.89)	<0.001

C-statistic was 0.815.

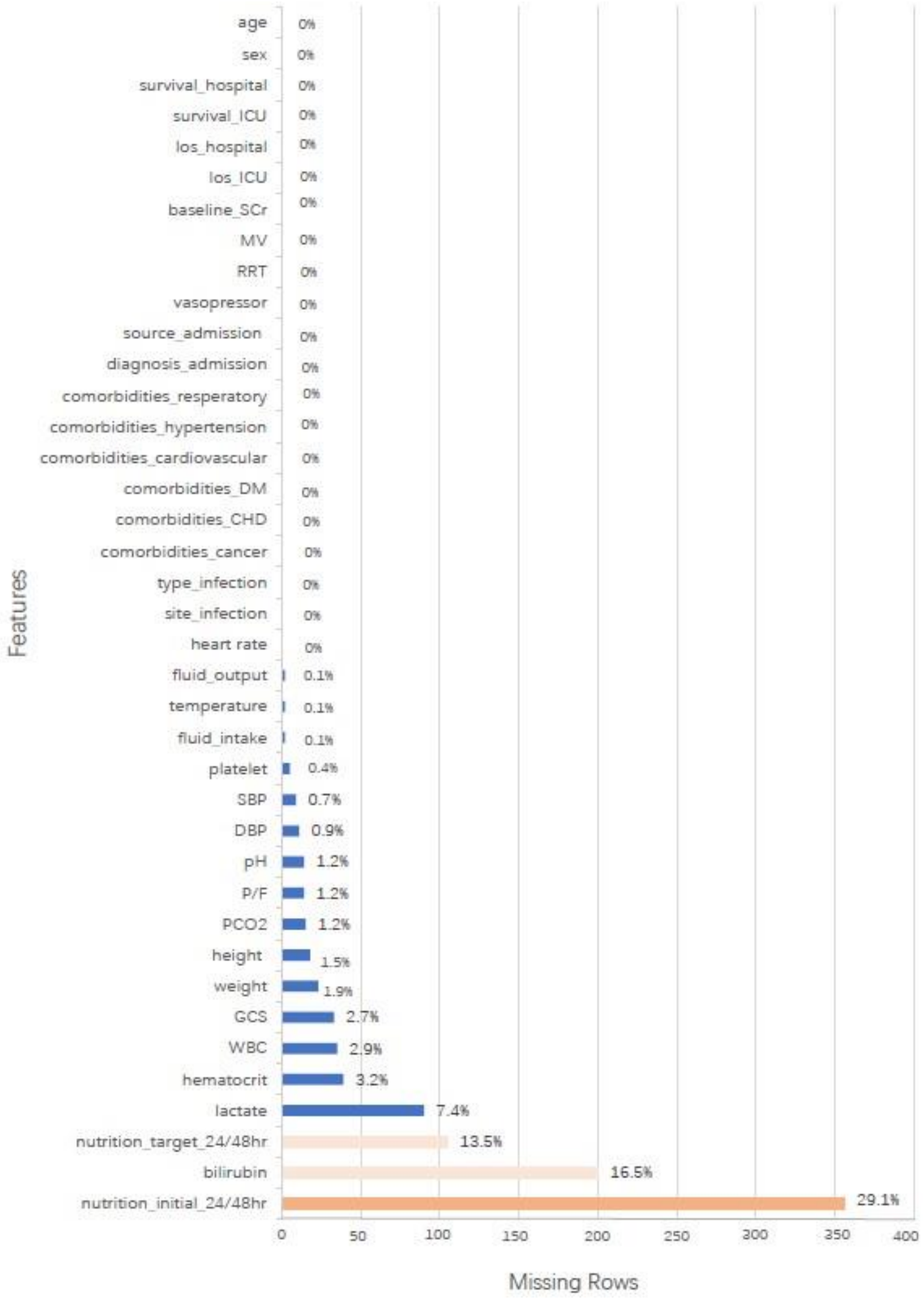

Fig S1. Missing rate for clinical and laboratory variables extracted from the database. Variables with missing rate greater than 30% were excluded from analysis.

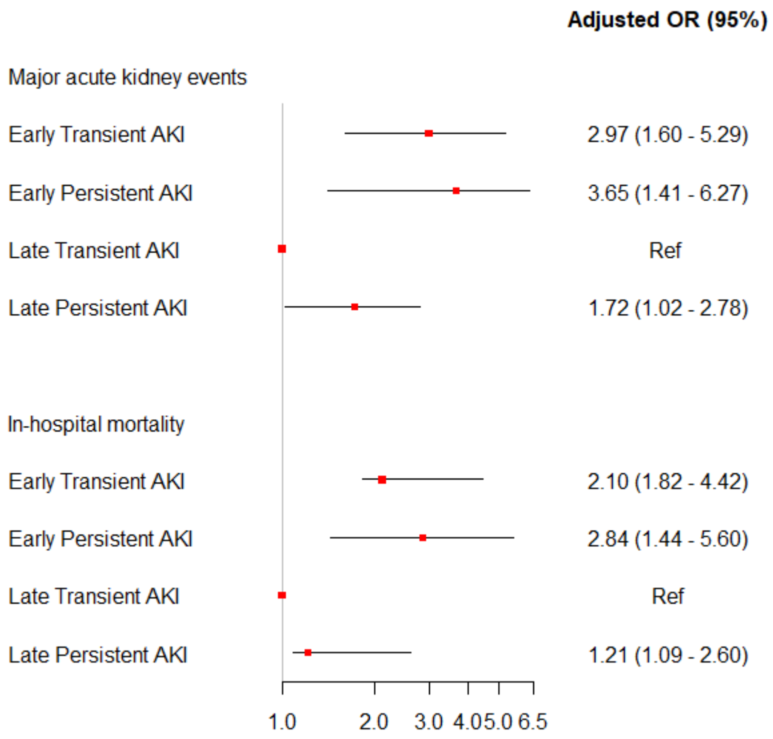

Fig S2. The association between AKI onset, evolution and clinical outcomes in BAKIT.
