# Supplementary material for: The role of lung ultrasonography in the assessment of overhydration in maintenance hemodialysis patients
Source: Ren Fail. 2023 Jan 17;44(1):1985–92. doi: 10.1080/0886022X.2022.2132169 (PMC9848227; doi:10.1080/0886022X.2022.2132169)
Supplement: Supplemental Material [file IRNF_A_2132169_SM3135.zip › IRNF 2132169/Additional_file_2.pdf]

Additional File 2

All other ethical bodies that approved our study in the various centers involved:

1. The institutional review board of West China Hospital, Sichuan University;
2. The institutional review board of Peking Union Medical College Hospital;
3. The institutional review board of Guangdong General Hospital;
4. The institutional review board of The First Affiliated Hospital of China Medical University;
5. The institutional review board of ZhongShan Hospital, FuDan University;
6. The institutional review board of The First Hospital of Jilin University;
7. The institutional review board of China-Japan Friendship Hospital;
8. The institutional review board of Beijing Friendship Hospital, Capital Medical University;
9. The institutional review board of Beijing Chaoyang Hospital, Capital Medical University;
10. The institutional review board of General Hospital of Ningxia Medical University;
11. The institutional review board of Xiangya Hospital, Central South University;
12. The institutional review board of Beijing Tongren Hospital, Capital Medical University;
13. The institutional review board of Peking University Third Hospital;
14. The institutional review board of Xuanwu Hospital, Capital Medical University;
15. The institutional review board of Beijing Tiantan Hospital affiliated to Capital Medical University.
